# High-Intensity Interval Training for Heart Failure Patients With Preserved Ejection Fraction (HIT-HF)-Rational and Design of a Prospective, Randomized, Controlled Trial

**DOI:** 10.3389/fphys.2021.734111

**Published:** 2021-09-24

**Authors:** Benedikt A. Gasser, Maria Boesing, Raphael Schoch, Stefanie Brighenti-Zogg, Julia M. Kröpfl, Elke Thesenvitz, Henner Hanssen, Jörg D. Leuppi, Arno Schmidt-Trucksäss, Thomas Dieterle

**Affiliations:** ^1^Department of Sport, Exercise and Health, Division of Sport and Exercise Medicine, University of Basel, Basel, Switzerland; ^2^Faculty of Medicine, University of Basel, Basel, Switzerland; ^3^University Department of Medicine, Cantonal Hospital Baselland, Liestal, Switzerland; ^4^Division of Cardiology, Clinic Arlesheim AG, Arlesheim, Switzerland

**Keywords:** diastolic heart failure, exercise capacity, training intensity, maximal oxygen uptake, strength-endurance training, quality of life

## Abstract

**Background:** The pathophysiology of HF with preserved ejection fraction (HFpEF) has not yet been fully understood and HFpEF is often misdiagnosed. Remodeling and fibrosis stimulated by inflammation appear to be main factors for the progression of HFpEF. In contrast to patients with HF with reduced ejection fraction, medical treatment in HFpEF is limited to relieving HF symptoms. Since mortality in HFpEF patients remains unacceptably high with a 5-year survival rate of only 30%, new treatment strategies are urgently needed. Exercise seems to be a valid option. However, the optimal training regime still has to be elucidated. Therefore, the aim of the study is to investigate the effects of a high-intensity interval (HIT) training vs. a moderate continuous training (MCT) on exercise capacity and disease-specific mechanisms in a cohort of patients with HFpEF.

**Methods:** The proposed study will be a prospective, randomized controlled trial in a primary care setting including 86 patients with stable HFpEF. Patients will undergo measurements of exercise capacity, disease-specific blood biomarkers, cardiac and arterial vessel structure and function, total hemoglobin mass, metabolic requirements, habitual physical activity, and quality of life (QoL) at baseline and follow-up. After the baseline visit, patients will be randomized to the intervention or control group. The intervention group (*n* = 43) will attend a supervised 12-week HIT on a bicycle ergometer combined with strength training. The control group (*n* = 43) will receive an isocaloric supervised MCT combined with strength training. After 12 weeks, study measurements will be repeated in all patients to quantify the effects of the intervention. In addition, telephone interviews will be performed at 6 months, 1, 2, and 3 years after the last visit to assess clinical outcomes and QoL.

**Discussion:** We anticipate clinically significant changes in exercise capacity, expressed as VO_2peak_, as well as in disease-specific mechanisms following HIT compared to MCT. Moreover, the study is expected to add important knowledge on the pathophysiology of HFpEF and the clinical benefits of a training intervention as a novel treatment strategy in HFpEF patients, which may help to improve both QoL and functional status in affected patients.

**Trial registration:** ClinicalTrials.gov, identifier: NCT03184311, Registered 9 June 2017.

## Introduction

Chronic heart failure (HF) is a common symptom complex characterized by shortness of breath, fatigue, fluid retention and severe exercise intolerance. It is estimated that about 1–2% of the adult population in developed countries suffer from this disease, among people over 70 years even more than 10% (Kuznetsova et al., 2009). In Europe, about 5% of hospital admissions in internal and geriatric medicine are caused by HF with a mean hospital stay of 11 days (Cleland et al., 2003). A study including 197 countries found that the costs of HF amounted to ~108 billion US Dollars per year globally (Cook et al., 2014).

Recent data show that about 50% of all HF patients have normal or near-normal left ventricular ejection fraction – a condition named HF with preserved ejection fraction (HFpEF) (Owan et al., 2006; Maeder and Kaye, 2009). Standardized diagnostic criteria for HFpEF became available for the first time with the most recent guidelines for diagnosis and treatment of acute and chronic HF published by the European Society of Cardiology in 2016 (Ponikowski et al., 2016). HFpEF is defined as a complex of signs and symptoms typical for HF: left ventricular ejection fraction >50%, elevated levels of brain natriuretic peptides (BNP > 35 pg·ml^−1^ and/or NT-proBNP > 125 pg·ml^−1^), and objective evidence of structural heart disease and/or diastolic dysfunction usually provided by echocardiography. A close association of HFpEF has been found with increasing age, female sex, obesity, arterial hypertension, ischemic heart disease, diabetes mellitus, and atrial fibrillation (Owan et al., 2006; Kuznetsova et al., 2009; Miljkovik and Spiroska, 2015). However, the pathophysiology of HFpEF has not yet been fully understood and symptoms are often misdiagnosed (e.g., as pulmonary edema of unknown origin) (Gupte and Hamilton, 2016). This is aggravated by the fact that many healthcare providers are still unaware of this form of HF (Oktay and Shah, 2015).

While the prognosis of patients with HFpEF is better than in HF with reduced ejection fraction (HFrEF) (Meta-Analysis Global Group in Chronic Heart, 2012; Lam et al., 2018) and has significantly improved over the last decades, medical treatment has been shown only to be successful to a small extent in improving prognosis in patients with HFpEF (Burkhoff, 2012; Solomon et al., 2012; Pitt et al., 2014). Current therapeutic standard approaches in HFpEF are still limited to relieving HF symptoms, e.g., by diuretics (Ponikowski et al., 2016). Since mortality in patients with HFpEF remains unacceptably high with a 5-year survival rate of 30%, new therapeutic strategies to beneficially affect prognosis in this steadily growing and high-risk patient group are urgently needed (Burkhoff, 2012).

Severe exercise intolerance is a hallmark of HF and a strong determinant of morbidity and mortality (Haykowsky and Kitzman, 2014). As a consequence of exercise intolerance, patients frequently adopt a sedentary lifestyle further aggravating signs and symptoms of HF. In this respect, cardiac rehabilitation aims to promote physical activity and increase patients' exercise capacity, which may enhance their quality of life (QoL) and clinical prognosis (Haykowsky et al., 2012). Several studies have demonstrated that aerobic training improves peak oxygen uptake (VO_2peak_) (Kitzman et al., 2010; Edelmann et al., 2011; Smart et al., 2012), diastolic function and oxygen delivery to and utilization by exercising muscles in patients with HFpEF (Fu et al., 2016).

In the process of designing training protocols, the optimal selection of training intensity plays a central role (Vanhees and Stevens, 2006) and is guided by parameters such as VO_2peak_, peak heart rate (HR_peak_), and ventilatory threshold (VT) assessed during an incremental exercise test (Binder et al., 2008).

While aerobic exercise is well-established for the treatment of HFrEF, no consensus exists for the management of HFpEF (Pina et al., 2003). For patients with HFrEF, a recent meta-analysis found a superior effect of high-intensity interval training (HIT) on VO_2peak_ compared to moderate-intensity continuous training (MCT) (Ramos et al., 2015). Furthermore, HIT was shown to be more effective than MCT in improving diastolic and endothelial dysfunction in HFrEF, which may also be relevant for patients with HFpEF (Wisloff et al., 2007; Ellingsen et al., 2017).

Despite the superiority of HIT over MCT in patients with HFrEF, no differences were found between HIT and MCT in the OptimEx trial that compared the effects of these training modalities in patients with HFpEF. However, patients in the MCT group completed five training sessions per week compared to three training sessions in the HIT group. Moreover, training modalities were not balanced between groups with regards to energy consumption and weekly exercise time in the MCT group, which were about 80% higher in the MCT group than in the HIT group (Mueller et al., 2021; Pandey and Kitzman, 2021). A further aspect to consider when judging the effects of HIT vs. MCT relates to the training intensity achieved during the course of a study compared to protocol targets. E.g., in SMARTEX-HF (Ellingsen et al., 2017), investigating the effects of HIT vs. MCT in patients with HFrEF, the HIT group trained less intensively while the MCT group trained more intensively than planned per study protocol. Thus, despite the fact that no differences between HIT and MCT were found in a well-designed trial such as OptimEx, a final conclusion in the optimal training regime in HFpEF patients and optimal exercise intensity cannot be drawn yet, since two relatively small studies indicate that HIT might be superior to MCT in patients with HFpEF. Angandi et al. investigated the effects of HIT vs. MCT in 15 patients with HFpEF and found a larger improvement for HIT compared to MCT. Four weeks of HIT significantly improved VO_2peak_ and left ventricular diastolic dysfunction, while no changes were observed in the control group following MCT (Angadi et al., 2015). Donelli da Silveira et al. found a superior effect of HIT on VO_2peak_ and diastolic function in 19 patients undergoing HIT or MCT (Donelli Da Silveira et al., 2020). A Norwegian study investigated the effects of a 6-month home training after 4 weeks of supervised exercise in 51 patients following coronary bypass surgery. The HIT group further increased VO_2peak_ by home exercise, whereas in the MCT group exercise capacity remained unchanged. The authors concluded that intense programs might provide an educational setting and a “kick-start” for changing activity patterns beyond what is obtained through outpatient rehabilitation (Donelli Da Silveira et al., 2020). HIT not only has been shown to be well-tolerated and safe in the majority of studies (Wisloff et al., 2007; Angadi et al., 2015; Ellingsen et al., 2017; Donelli Da Silveira et al., 2020), but might also be the most promising approach to induce beneficial effects on macro- and microvascular structures (Konigstein et al., 2021), at least in HFpEF. In order to make this study as safe as possible for this patient group we decided for a gradual intensity increase during the intervention.

VO_2peak_ is a valid and reproducible marker for exercise capacity and a strong predictor of prognosis and QoL (Haykowsky et al., 2012). In the HF-ACTION Trial, a 6% increase in VO_2peak_ was associated with a 5% lower risk of all-cause mortality or all-cause hospitalization. Furthermore, patients with an improvement in VO_2peak_ of <2 ml min^−1^ kg^−1^ after exercise training had a lower event-free survival compared to good responders (Caminiti et al., 2016).

Cardiac and vascular remodeling and fibrosis stimulated by chronic inflammation appear to be among the most important factors for the progression of HFpEF (Ponikowski et al., 2016). Biomarkers related to macro- and microvascular changes, cardiac hypertrophy, fibrosis and endothelial dysfunction may provide further insight into the pathogenesis and prognosis of HFpEF. Furthermore, the impact of the hematopoietic system on total physical performance has to be considered (Mancini et al., 2003). In fact, several studies suggest that total hemoglobin mass (tHb-mass) instead of Hb-serum concentration may be the major determinant of exercise capacity in HF (Miller and Mullan, 2015; Cattadori et al., 2017; Otto et al., 2017; Montero et al., 2019).

In addition to exercise testing, monitoring of habitual physical activity may offer further useful information to assess functional status, as it accounts for changes in activity behavior, which exercise testing does not (Jehn et al., 2009). Fast walking determined by accelerometry has been shown to be an excellent predictor for discriminating patients with HF in different New York Heart Association (NYHA) functional classes (Jehn et al., 2009).

### Objectives

The primary aim of this study is to investigate the impact of a supervised 12-week HIT on exercise capacity, measured as VO_2peak_, in patients with HFpEF, compared to an MCT. As secondary objectives, training-related effects on biomarkers as surrogates of systemic inflammation, endothelial function and clinical prognosis, cardiac and vascular structure and function, functional status, QoL, body composition, and habitual physical activity will be examined. An additional secondary objective is to examine the tHb-mass with regards to VO_2peak_.

## Methods and Analysis

### Study Design and Setting

The proposed study is a prospective, 2-arm randomized controlled trial. The intervention arm includes the investigation of a supervised 12-week HIT on exercise capacity, functional status and QoL in patients with HFpEF. A control group training with isocaloric MCT will serve as comparator. Patients will be randomized in a 1:1 ratio to either the intervention or control group using the computer-based system provided by Castor. Based on a sample size calculation, a total of 86 patients will be enrolled (43 patients in each group). The Department of Sport, Exercise and Health (DSBG) at the University of Basel will be the main study center collaborating with the Division of Cardiology at the Clinic Arlesheim, local general practitioners and cardiologists.

### Recruitment

Members of the study team will screen the medical files of the DSBG and the Clinic Arlesheim, Division of Cardiology, for previously treated patients with an established diagnosis of HFpEF. Eligible patients will be contacted by phone and asked whether they are willing to take part in this study. If patients agree to participate, they will be invited to the DSBG for further information, giving informed consent, and initiating the study procedures. In addition, collaborating institutions will also provide patients with HFpEF with an information sheet about the goal and the conduct of the present study and refer them to the DSBG for final screening. In order to minimize bias that may be introduced by differences in executing and interpreting technical exams, questionnaires, or training sessions, all study related measurements and procedures will be performed in a standardized manner at the DSBG. Inclusion and exclusion criteria are presented in Table 1.

**Table 1:** Inclusion and exclusion criteria.

**Inclusion criteria**	**Exclusion criteria**
Informed consent as documented by signature	Planned cardiac interventions in the following 6 months
NYHA functional class II-III	Unstable angina pectoris
Signs and symptoms of chronic HF:	Uncontrolled brady- or tachyarrhythmia
Dyspnea	Uncontrolled hypertonic blood pressure
Paroxysmal nocturnal dyspnea	Severe uncorrected valvular heart disease
Reduced exercise capacity	Paroxysmal atrial fibrillation
Extended recovery after exercising	Clinically significant concomitant disease states (e.g., advanced renal failure, hepatic
Fatigue	dysfunction, insulin-dependent diabetes)
Peripheral edema (lower leg, ankle)	
EF > 50%	COPD grades III-IV according to the GOLD classification
Structural or functional changes in echocardiography:	
LAVI > 34 ml·m^−2^ OR	On-going cancer treatment
LVMI > 115 g·m^−2^ (men), >95 g·m^−2^ (women) OR	Significant musculoskeletal disease limiting exercise capacity
E/E' ratio >13 AND mean E' septal and lateral wall <9 cm·s^−1^	
NT-proBNP > 125 pg·ml^−1^	Active infection
At least 4 weeks on stable medical treatment or without signs and symptoms of cardiac de-compensation	Immunosuppressive medical therapy
	Blood transfusion within the previous 30 days
Trainable:	Pregnancy or lactation
VT > 40% of predicted VO_2max_ AND	
VO_2peak_ > 10 ml·min^−1^·kg^−1^ at the screening visit	Known or suspected non-compliance, drug or alcohol abuse
	Inability to follow the procedures of the study, e.g., due to insufficient language skills, psychological disorders, dementia, etc.
	Participation in another intervention study
	Enrolment of the investigators, their family members, and other persons involved in the study procedures
	Life-expectancy <6 months
	Age <18 years

*COPD, Chronic obstructive pulmonary disease; E/E' ratio, Ratio between mitral peak velocity of early filling (E) to early diastolic mitral annular velocity (E'); EF, Ejection fraction; GOLD, Global initiative for chronic obstructive lung disease; HF, Heart failure; LAVI, Left atrial volume index; LVMI, Left ventricular mass index; NT-proBNP, N-terminal prohormone of brain natriuretic peptide; NYHA, New York Heart Association; TSAT, Transferrin saturation; VO_2max_, Maximal aerobic capacity; VO_2peak_, Peak oxygen uptake; VT, Ventilatory threshold*.

### Description of the Study Procedures

**Figure 1:**
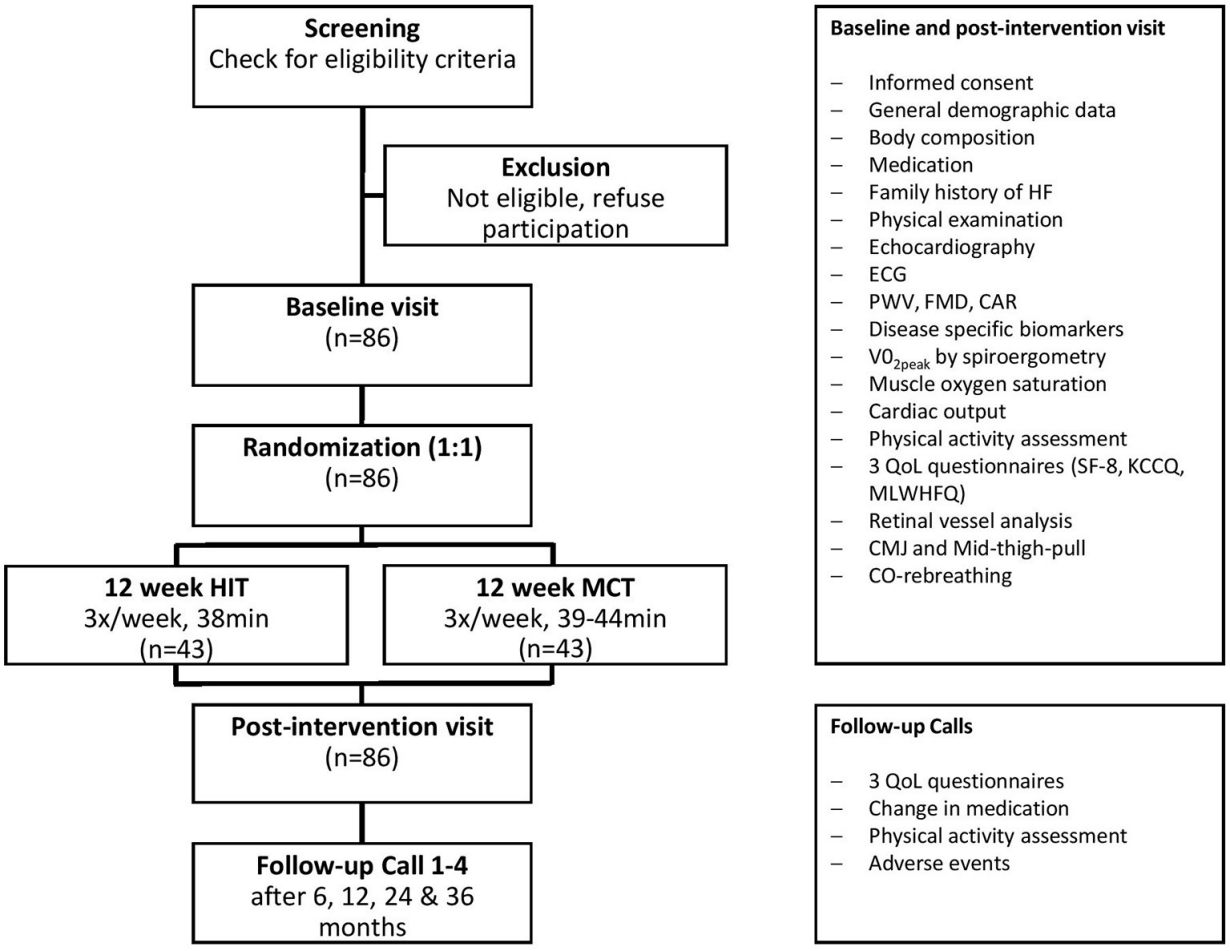
Study organization. Flow chart for patients, showing screening, inclusion and exclusion, randomization, intervention, tests at baseline, post-intervention visit and follow-up. HIT, high-intensity interval training; MCT, moderate continuous training; HF, heart failure; ECG, electrocardiogram; PWV, pulse wave velocity; FMD, flow mediated dilation; CAR, carotid artery reactivity; VO_2peak_, maximal oxygen uptake; QoL, quality of life; SF-8, short form health survey 8; KCCQ, Kansas City cardiomyopathy questionnaire; MLWHFQ, Minnesota living with heart failure questionnaire; CMJ, counter movement jump.

The study procedures and assessments are shown in Figure 1. Patients will undergo a screening for eligibility. If inclusion criteria are fulfilled, a baseline visit and a post-intervention visit will follow. First, general demographic data will be recorded, an electrocardiogram, echocardiography, several laboratory tests, measurement of body composition, pulse wave velocity (PWV) and flow mediated dilation (FMD) will be conducted, and questionnaires concerning QoL [8-Item Short Form Health Survey (SF-8), Kansas City Cardiomyopathy Questionnaire (KCCQ) and Minnesota Living With Heart Failure Questionnaire (MLWHFQ)] will be filled in by the patients. A urinary sample will be collected. A spiroergometry including a pre- and post-exercise blood collection will be performed to determine VO_2peak_, and an accelerometer (wrist) will be handed out to document habitual physical activity over the 14 subsequent days. If patients do not fulfill the inclusion criteria at the screening visit, they cannot continue to participate in this study. Patients included and randomized to the HIT group will then start to perform a 12-week high-intensity interval training (HIT) on a bicycle ergometer combined with strength training (3 sessions per week) in the Division of Physical Therapy at the DSBG supervised by sports scientists of the study team. Patients in the control group will undergo a moderate continuous training (MCT) together with strength training. After the last training session, the study measurements will be repeated in all patients (intervention and control group) within the following week in order to monitor the effects of the intervention (post-intervention visit). Total study duration including the 3 study visits will be 3 months for each patient. Furthermore, at 6 months, 1, 2, and 3 years after the last study visit, telephone interviews will be conducted to assess clinical outcomes (change in medication, adverse events), physical activity assessment, and QoL (Figure 1). In addition to each follow-up call, the wrist accelerometer will be sent to the patients together with a postage-paid return envelope and patients will be asked to wear it for 8 days. As general recommendation, all patients will be asked to follow a healthy diet according to the Swiss Society for Nutrition and maintain their standard medication and diet during the intervention. Standard medical procedures will remain in the responsibility of the individual primary care physician, respectively, cardiologist.

### Primary Outcome

The primary outcome will be the change in VO_2peak_ as a marker for exercise capacity from the baseline visit to the end of the 12-week training intervention (post-intervention visit) following HIT with strength training compared to MCT with strength training. VO_2peak_ will be determined by spiroergometry. Patients will undergo an incremental symptom-limited exercise test on an electronically operated bicycle ergometer (Ergoselect 200, Ergoline, Bitz, Germany) using one of two fixed ramp protocols, depending on the patient's fitness status (protocol 1: warm-up unloaded, then increase of 7 W·min^−1^; protocol 2: warm-up at 10 W, then increase of 10 W·min^−1^) (Task Force of the Italian Working Group on Cardiac et al., 2006). The test will be performed in an temperature-controlled laboratory under non-fasting conditions. After the 3-min warm-up, patients will be instructed to pedal at a constant rate of 60 rpm until exhaustion or until signs of ischemia or serious cardiac arrhythmias appear. Exhaustion will be defined as a respiratory exchange ratio (VCO_2_/VO_2_) >1.05 (Wagner et al., 2020). The exercise bout will be followed by a cool-down at a maximum of 25 W for 3 min. VO_2peak_ will be defined as the highest value reached during exercise. The ventilated air volume and expired gas concentrations will be measured via breath-by-breath analysis (MetaMax 3B, Cortex Biophysik GmbH, Leipzig, Germany). In addition, heart rate will be measured with a 12-channel electrocardiography (Custo Med GmbH, Ottobrunn, Germany). Calibration of volume and gases will be performed before each test.

### Secondary Outcomes

Secondary outcomes measured at baseline and follow-up will include the assessment of

**Figure 2:**
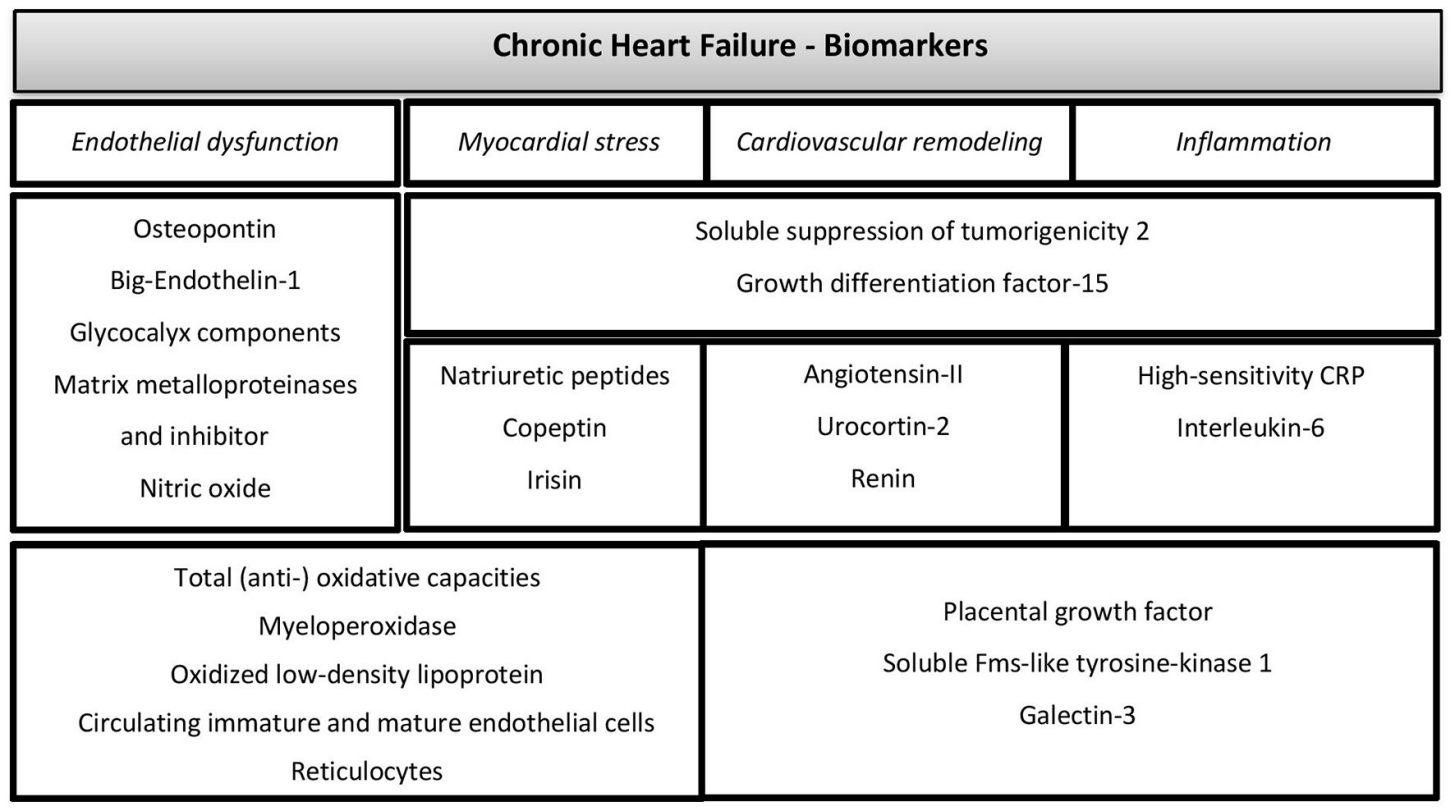
Overview of blood biomarkers. Laboratory parameters reflecting biomarkers of chronic heart failure; CRP, C-reactive protein.

- tHb-mass by the optimized carbon monoxide (CO) rebreathing method. The measurement procedure includes inhaling a small amount of CO using the Detalo Performance^™^-rebreathing device (Detalo Health Aps, Denmark). CO completely binds to Hb in the blood, which enables the calculation of tHb-mass from the difference of CO-Hb concentration before and after inhalation (~5%) in capillary blood. Two to three capillaries will be taken at each time point and examined with a blood gas analyzer (Montero et al., 2019). CO-Hb levels below 10% are usually not associated with adverse symptoms and any CO-Hb increase should already be reduced to 50% within 2 h after rebreathing cessation (Oberholzer et al., 2020).
- NT-proBNP as a prognostic factor and the determination of the correlation of VO_2peak_ and NT-proBNP. NT-pro-BNP will be analyzed on a cobas system (Roche Dignostics, Rotkreuz, Switzerland).
- Further disease-specific biomarkers such as renin, angiotensin-II, urocortin-2, osteopontin, soluble suppression of tumorigenicity-2, galectin-3, growth differentiation factor-15, copeptin, big-endothelin-1, placental growth factor/soluble Fms-like tyrosine-kinase-1, high-sensitivity C-reactive protein, interleukin-6, insulin-like growth factor-binding protein-7, irisin, glycocalyx components, matrix metalloproteinases, -activity and inhibitor, nitric oxide, total (anti-) oxidative capacities, myeloperoxidase, oxidized low-density lipoprotein, circulating immature and mature endothelial cells, and reticulocytes (see Figure 2. for overview).
- All parameters will be analyzed in serum. Irisin and glycocalyx components such as syndecan-1, heparan sulfate and hyaluronan concentrations will be determined by enzyme-linked immunosorbent assay (ELISA) kits (Phoenix Pharmaceuticals Inc, Burlingame, USA; Diaclone Research, Besancon, France; Seikagaku, Tokyo, Japan; Fa. Cusabio Art.Nr.: CSB-E09585h; Echelon Biosciences, Salt Lake City, USA; respectively). Total (anti-) oxidative capacities as well as oxidized low-density lipoprotein will be determined using the Labor Diagnostika Nord (Nordhorn, Germany) assays. Parameters of endothelial matrix remodeling (matrix metalloproteinases, -activity, and inhibitor) and myeloperoxidase will also be determined by ELISAs (R&D Systems Europe, United Kingdom). MMP activity will be analyzed using Fluorogenic Peptide Substrate (R&D Systems Europe, United Kingdom). Circulating immature and mature endothelial cells and reticulocytes will be analyzed by flow cytometry (CytoFLEX, Beckman Coulter and ADVIA, Siemens, Switzerland, respectively).
- Echocardiographic parameters of the left ventricular structure, systolic and diastolic function measured using a Full HD Color Doppler Ultrasound Scanner UF-890AG (Fukuda Denshi, Tokyo, Japan) by experienced echocardiographers according to recent international guidelines and standards (Miljkovik and Spiroska, 2015; Oktay and Shah, 2015; Ponikowski et al., 2016). In addition, ventricular-arterial coupling defined as the ratio of arterial elastance and end-systolic elastance will be approximated echocardiographically according to the method described by Antonini-Canterin et al. (2013).
- Peak arterial-to-venous oxygen content difference (Da-vO_2_) calculated using the Fick Principle (Peak Da-vO_2_ = VO_2peak_/peak cardiac output) (De Cort et al., 1991).
- Brachial-ankle PWV measured oscillometrically in the supine position at the right and the left upper arm and ankles with the VaSera VS-1500N Vascular Screening System (Fukuda Denshi Co. Ltd, Tokyo, Japan). Central PWV calculated applying the ARCSolver algorithm (Wassertheurer et al., 2010), which has shown excellent comparability with tonometric measurement of central PWV (Endes et al., 2015).
- NYHA functional class.
- QoL assessed by the questionnaires SF-8 (Yiengprugsawan et al., 2014), KCCQ (Green et al., 2000; Van Den Berg-Emons et al., 2000) and MLWHFQ (Van Den Berg-Emons et al., 2000; Morcillo et al., 2007).
- Body composition will be analyzed by four-segment bioelectrical impedance analysis using the InBody 720 (Inbody Co. Ltd., Seoul, South Korea). Body mass index (BMI = weight/height^2^) and waist-to-hip ratio (WHR = waist circumference/hip circumference).
- Physical Activity Level (PAL), number of daily steps and time spent at different walking speeds (min·day^−1^) will be measured using a waterproof micro-electromechanical triaxial accelerometer worn on the non-dominant wrist (GeneActiv, Activinsights Ltd, Kimbolton, Cambridgeshire, UK) to assess physical activity intensity (light, moderate, vigorous) and periods of inactivity, sleep and wake over 7 days for 24 h per day (Esliger et al., 2011; Wijndaele et al., 2015; Doherty et al., 2017).
- Muscular strength measured with a so-called Mid-Thigh-Pull Test. This isometric test is conceptualized in order to rate whole body force; the force of all extensors of the leg e.g., M. rectus femoris and of the back such as M. erector spinae as well as muscles of the forearm and hand. The test is comparable to elevate a table by hand. Two factors will be measured: the ability of a patient to generate maximum force (‘Peak Force') and the second factor is measuring the increase of force over time (Rate of Force Development) (Scott et al., 2011). Furthermore, a strength measurement by a counter movement jump shall be performed on a force plate (Leonardo Mechanograph^®^, Novotec Medical, Pforzheim, Germany) to measure peak power. The instruction will be to jump with the head and chest as high as possible, thus producing the maximum elevation of the center of mass. Participants, who are unable to jump, will be instructed to push as fast and hard as possible in order to generate power on the plate. The most critical outcome parameter of this test is the maximum power output (peak power), which equals the force normalized to the body weight of the participant. The method has been validated in young and older adults (Maden-Wilkinson et al., 2015).
- Measurement of muscle oxygen saturation. Muscle oxygen saturation is measured with near-infrared spectroscopy (NIRS, Portamon, Artinis Medical Systems, Elst, The Netherlands). NIRS relies mainly on two characteristics of human tissue. First, the relative transparency of tissue to light in the NIR range, and second, the oxygenation-dependent light absorbing characteristics of Hb. By using a number of different wavelengths, the relative changes in Hb concentration can be displayed continuously and saturation, respectively, absorption can be measured. If the absorption is known, the Lambert-Beer law can be used to calculate the chromophore's absorption (Scholkmann et al., 2014). The technique on which NIRS relies is closely analogous to the technique of pulse oximetry and is in consequence not painful and non-invasive (Fluck et al., 2017; Fitze et al., 2019). The NIRS device is placed without pressure on the M. vastus lateralis half between trochanter major and joint gap of the knee. There are no structural changes in the underlying tissue and no space between the skin and the sensor where external light could penetrate. The skin is cleaned and shaved before the measurement. To keep the device in position, medical adhesive tape is used and a black bandage is tied around the thigh without pressure to protect the sensor from extraneous light.
- Cardiac output with cardiac impedance measurements using the Physioflow device (Physioflow, Manatec Biomedical, Poissy, France) (Endes et al., 2015). The measurement is based on a formula including heart rate, stroke volume and body surface area. Six pre-gelled electrodes will be placed on the thorax, which are connected to an electronic processing unit (2 on the left side of the neck, between earlobe and collarbone, 2 on the chest and 2 near the xiphisternum). After attaching the electrodes, the device is calibrated during 30 heart beats. Cardiac output will be measured continuously and be monitored from the beginning of the spiroergometry until the maximum workload is reached and the cool-down is completed.
- The measurement of flow-mediated dilation (FMD), a principle to measure the integrity of the endothelia. It refers to dilation of an artery when blood flow increases in that artery. The primary cause of FMD is release of nitric oxide by endothelial cells through shear stress. FMD of the brachial arteries provides a non-invasive alternative to other measurement procedures. To determine FMD, brachial artery dilation following a transient period of forearm ischemia is measured using ultrasound (UNEFEX 38G 3.0, UNEX Co., Nagoya, Japan) (Konigstein et al., 2021). The rationale behind the measurement is that endothelial cells are sentinels of cardiovascular health. Their function is reduced by the presence of cardiovascular risk factors, and is regained once pathological stimuli are removed (Charakida et al., 2013; Tomiyama et al., 2015).
- Static and dynamic retinal vessel analysis using the retinal vessel analysis system (IMEDOS Systems, Jena, Germany) and a fundus camera (450 FF; Carl Zeiss, Jena, Germany). We will take three valid static images and two dynamic videos of the retina to quantify retinal microvascular structure and function. Conventional eye drops (Tropicamide 0.5%) will be used for pupil dilation of one eye. Retinal vessel diameters will be calculated as central retinal arteriolar and venular equivalents. Three flicker cycles are applied and averaged to calculate the flicker light-induced dilatation in %change relative to baseline. Details of the standardized procedures have been published previously (Streese et al., 2018).
- Carotid artery reactivity (CAR), a parameter non-invasively assessed by transcutaneous ultrasound, to examine endothelial function following sympathetic stimulation produced by the cold pressor test. Right carotid artery diameter is recorded before and during 90 s of immersion of the hand up to the wrist in ice water (4°C). Images will be obtained using a high-resolution ultrasound machine (UF-760AG, 5-12 MHz linear array transducer, Fukuda Denshi Co. Ltd., Tokyo, Japan) (Liao et al., 2012).

### Adverse Events and Safety Considerations

In both groups (MCT and HIT) adverse events (AE) and serious AE will be recorded. All potential cardiovascular events will be considered to be serious AEs (e.g., atrial and ventricular arrhythmia, unstable angina pectoris, clinical worsening of HF requiring hospitalization or intensified diuretic therapy, cardiovascular death) and will be immediately treated, recorded and/or discussed in detail to plan further procedures. If clinically indicated, laboratory parameters (kidney and liver values, International Normalized Ratio, hematology, etc.) will be measured and the patient's personal cardiologist will be informed to discuss further procedures.

Safety and adherence after end of the study will be checked during follow-up calls. Patients will be referred to adequate long-term rehabilitation settings (e.g., ambulant cardiovascular rehabilitation, KARAMBA; or the Swiss Physical Activity Promotion in Primary Care System, PAPRICA).

### Randomization

Patients will be randomly allocated to either the intervention or the control group in a 1:1 ratio stratified by gender. Randomization will be done using a computer-based system implemented in the Castor software (Castor, Amsterdam, Netherlands). The randomization procedure includes a minimization algorithm. The randomization procedure is implemented in the online electronic-data-capture system provided by Castor. Only authorized study personnel will have access to the randomization tool and can assign a treatment to a new study patient. Due to the minimization algorithm, the allocation sequence is totally concealed until randomization is carried out.

### Blinding

It is not possible to blind patients or investigators performing the randomization and training sessions. Therefore, un-blinding procedures are not applicable. However, data analysis will be performed by investigators blinded for patient data and allocation to HIT, respectively, MCT.

### Intervention

**Figure 3:**
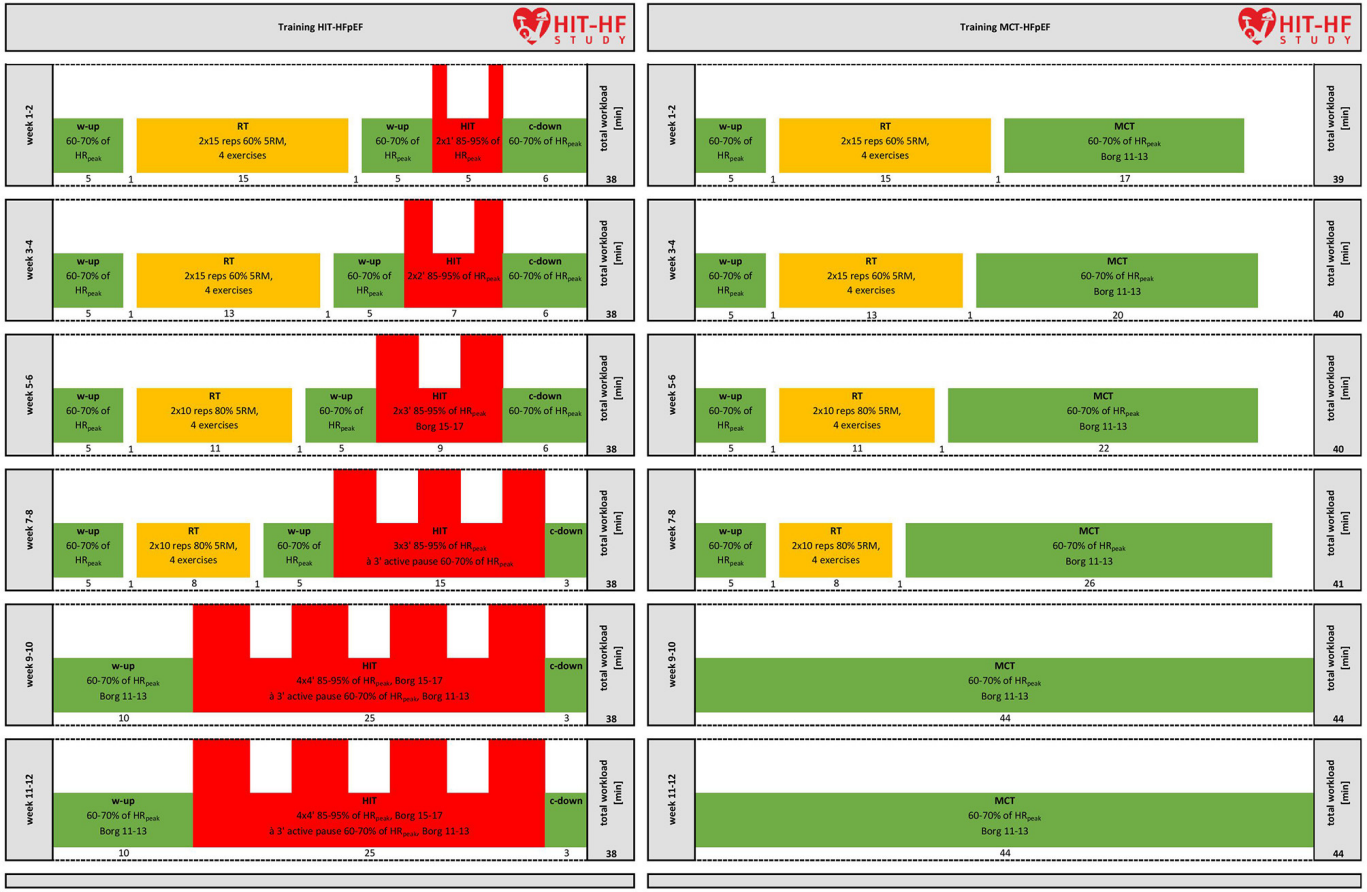
Overview of the endurance-strength training protocols. Training protocol for HIT and MCT during the 12-week intervention period. W-up, warm-up; HR_peak_, heart rate peak; 5RM, 5-repetition maximum; RT, resistance training; HIT, high-intensity interval training, c-down, cool-down; MCT, moderate continuous training.

All patients will perform a 12-week training program 3 times per week on a bicycle ergometer (Ergoselect 200, Ergoline, Bitz, Germany), Figure 3. During training, patients will wear the Sense system (CSEM, Neuchâtel, Switzerland). The Sense system measures heart rate, breathing rate and a 1-channel ECG, which can be displayed live on a tablet to control exercise intensity and heart rhythm. If heart rate is too high or too low, intensity is adjusted. The Borg 6-to-20 Scale will be used to assess the rate of perceived physical exertion during and after each training session. HIT and MCT are designed to achieve a similar energy expenditure (isocaloric design). Every training session will be supervised and controlled by sports scientists to keep patients train in the planned intensity in order to avoid overlap of training intensities of HIT and MCT.

#### HIT Group

During the 12-week intervention period, we will use an adapted version of the classical 4 x 4 min protocol of Wisloff et al. (2007) with increasing HIT interval frequency and duration from week 1–9 without any further increase until week 12. From week 1 to 8, resistance training based on the intervention study of Beckers et al. (2008) and recent evidence from literature review (Giuliano et al., 2017) with decreasing volume and current recommendations (Schindler et al., 2019) will be conducted: Since patients might not be able to sustain a traditional 4-min HIT protocol (Schindler et al., 2019), we decided to increase the intensity from week to week. Patients will warm up for 5 min at moderate intensity (60–70% of HR_peak_, Borg 11–13) before resistance training and 5 min before cycling intervals with increasing time from 1 to 4 min and increasing frequency from 2 to 4 intervals at high intensity (85–95% of HR_peak_, Borg 15–17). Each interval will be separated by a 3 min active pause at 60–70% of HR_peak_ (Borg 11–13). The training session will end with a 3 min cool-down at moderate intensity (60–70% of HR_peak_). Resistance training will consist of four strength exercises (Leg Press, Leg Curl, Leg Extension, and Calf Raises) on weight machines (Cybex, divison of Lumex Inc, NY, USA), with 2 × 15 repetitions per set at 60% of the five repetition maximum (5RM) from week 1 to 4 and 2 × 10 repetitions per set at 80% of the 5RM from week 5 to 8. There will be no resistance training from week 9 to 12. Total exercise time will be 38 min. Resistance exercise shall be performed in order to prepare for the intensive endurance parts. Interference effects of strength before endurance training seem negligible for subjects with HF (Hickson, 1980; Coffey and Hawley, 2017; Vechin et al., 2021).

#### MCT Group

Patients perform the same strength exercises and then cycle continuously over a period of 12 weeks, increasing from initial 17 to 26 min from week 1 to 8 and 44 min from week 9 to 12 at 60–70% of the HR_peak_.

### Criteria for Modifying or Discontinuing the Intervention

Workload of the bicycle ergometer will be continuously adjusted in order to maintain a constant relative exercise intensity throughout the 12-week training period according to the protocol (Wisloff et al., 2007). Termination criteria include intolerable symptoms during exercise training [e.g., severe transient dyspnoea, dizziness, systolic blood pressure >260 mmHg and/or diastolic blood pressure >115 mmHg, frequent arrhythmias, palpitations (>30 s)] as well as subjective physical or mental overload (e.g., strong discomfort, excessive stress reactions, fatigue), which prevent the patient from further participation.

Based on the current epidemiological situation and regulations in Switzerland, it seems very likely to be able to offer continued training during another COVID-lockdown.

### Compliance With the Study Intervention

Good compliance will be ensured by friendly research staff, motivating training units and improving health outcomes, such as better QoL. In case a patient will miss a training session, it will be rescheduled, if possible. A compliance of 80% (29 sessions) will be required to be considered in the analysis of the per-protocol set (PPS).

### Concomitant Treatments

Concomitant treatments or medication considered necessary by the treating physicians are permitted. However, if the ability to exercise is affected in case of worsening HF (e.g., cardiac decompensation, NYHA IV) or any new diagnosis of concomitant disease (e.g., cancer, pulmonary hypertension, trauma, and so on), patients will be excluded from the study and will not be allowed to resume the study procedures at a subsequent date.

### Sample Size

We use a closed form formula to assess the sample size, as implemented in the R function “power.t.test()”. This provides us a sample size for comparison of two independent groups. Borm et al. (2007) showed that including the baseline value as a covariate in an ANCOVA design as planned here, can reduce the required sample size while maintaining power. Accordingly, the required sample size would be N_total_ = N_t−test_^*^ (1 – ρ^2^) + 2, where ρ is the within patient correlation between baseline values and follow-up. To obtain realistic assumptions on the baseline VO_2peak_ distribution required for the sample size calculation, we performed a meta-analysis of five research papers (Kitzman et al., 2010, 2013; Edelmann et al., 2011; Alves et al., 2012; Smart et al., 2012). The fixed effects meta-analysis suggested the standard deviation of VO_2peak_ at baseline to be 3.39 ml min^−1^ kg^−1^. For the sample size estimation, we take a slightly more conservative value, assuming baseline VO_2peak_ to have a standard deviation of 4.2 ml min^−1^ kg^−1^, ~25% higher than suggested by the meta-analysis. In addition, we assume the within-patient correlation to be 0.7 ml min^−1^ kg^−1^, which is rather low for such measurements. We set the type-I error rate, α, to 0.05, and the power of the study (1-β) to 80%. We only allow a dropout rate of 10% for the primary outcome at 12 weeks.

Under these assumptions, with a 1:1 treatment allocation ratio, 86 patients need to be recruited in order to show a minimal clinically relevant difference of 2 ml min^−1^ kg^−1^, while maintaining power for the PPS with 77 evaluable patients.

### Statistical Methods and Data Analysis

The full analysis set (FAS) will include all patients randomized to the trial, regardless of compliance. The PPS will include all patients from the FAS, who had a 12-week post-intervention visit and who complied with their assigned treatment.

The primary analysis will be done on the FAS and based on the intention to treat principle. Patients will be analyzed according to the group to which they were assigned. Missing values in the primary endpoint will be imputed using multiple imputations via chained equations based on baseline characteristics. An ANCOVA approach will be taken: a linear regression model will be used to model patient VO_2peak_ at post-intervention as outcome, with the baseline value as an independent covariate. As an explorative analysis, the association of tHb-mass and patient type, as well as their combined effects on VO_2peak_ will be examined using multiple regression models or other methods for estimating causal effects. As a safety analysis, the number and type of AEs will be summarized and reported per group. In addition, the number of non-compliant patients and their baseline characteristics compared between treatment groups will be summarized.

Confounding variables such as baseline patient characteristics (e.g., age, BMI, smoking/drinking), comorbidities, medication, and baseline fitness level will be accounted for during regression analyses.

### Data Collection, Management, and Retention

Data will be anonymized and stored in a pseudonymized manner by using the patient IDs. Personal data and contact information for the follow-up phone calls and assessment of physical activity will be entered into a separate excel file with restricted access for staff performing these calls. Collection and management of the clinical trial data will be done using an electronic data capture (EDC) system based on the software Castor. Source data, randomization and pseudonymization lists will be kept under lock and key. Password protection ensures that only authorized persons can enter the system to view, add or edit data according to their permissions. The EDC system will be locked after all data have been monitored and all raised queries have been resolved. Data will be archived at the DSBG. Independently from investigators, regulatory authorities can audit this trial. Direct access to source documents will be permitted for purposes of monitoring, audits and inspections. All involved parties must keep the participant data strictly confidential. Any results of this study will be published in an anonymized manner. The study protocol and dataset shall be accessible to any regulatory authority after publication for at least 10 years.

## Discussion

Heart failure, in particular heart failure with preserved ejection fraction (HFpEF) constitutes a large and increasing burden to the national and international healthcare systems. Nevertheless, the etiology of HFpEF has not yet been fully understood and symptoms are often misdiagnosed. Since mortality in patients with HFpEF remains unacceptably high with a 5-year survival rate of only 30%, new therapeutic strategies are urgently needed (Burkhoff, 2012; Meta-Analysis Global Group in Chronic Heart, 2012; Lam et al., 2018). Maximum oxygen uptake (VO_2peak_) as a measure for exercise capacity is a strong and independent predictor of morbidity and mortality in patients with HFpEF, but few data are available on the efficacy of training programs (Fukuta, 2020). A study of Angadi et al. (2015) found that HIT significantly better improved VO_2peak_ and left ventricular diastolic dysfunction in HFpEF patients compared to MCT. They also demonstrated that HIT was well-tolerated in this cohort consisting predominantly of female older and overweight or even obese individuals. The study of Donelli Da Silveira et al. (2020) (*n* = 19, 12-week duration) that investigated a relatively young sample (mean age: 60 years) with few comorbidities supports these findings. However, in OptimEx, the largest study conducted in HFpEF patients to date comparing MCT vs. HIT, HIT was not superior over MCT (Mueller et al., 2021; Pandey and Kitzman, 2021). However, the results of OptimEx should be interpreted with caution. Patients in the MCT group completed five training sessions per week compared to three training sessions in the HIT group and training modalities were not balanced between groups with regards to energy consumption, considerably limiting the generalizability of the results. These findings are supported by a recent meta-analysis (Fukuta et al., 2019). Besides the effects on the cardiopulmonary system, training directly affects the skeletal muscle (Hoppeler et al., 1985, 2011; Fukuta et al., 2019). Results from a recent study indicate that resistance training - even as a single intervention - can increase muscle strength, aerobic capacity and QoL in patients with HF (Giuliano et al., 2017). Furthermore, a not-too-strenuous aerobic training may be better suited for subjects at low training states such as HFpEF patients because of better coping with low aerobic training stimuli (Schindler et al., 2019) – as e.g., in the beginning of our training regime. Introducing strength training at the beginning might not only allow to prepare skeletal muscle to adapt to a constantly improving cardiopulmonary system, but also to start with a lower aerobic stimulus, while still having a reasonable total stimulus per training unit (Fluck et al., 2017; Fitze et al., 2019). In consequence, a combination of strength and endurance training might be better suited for patients with HFpEF (Ponikowski et al., 2016; Schindler et al., 2019).

Our analyses of biomarkers reflect the most important pathogenetic pathways, will provide further important insights into the pathophysiology of HFpEF, and might be helpful in predicting the response to exercise therapy in order to effectively guide risk stratification as well as preventive and therapeutic measures.

## Trial Status

The trial will be conducted according to the protocol version 6 of 20th of June 2020. Patient recruitment started in September 2020 and is expected to run consecutively until November 2022. Data collection of the intervention phase is expected to be completed in February 2023 and data analysis in May 2023. Data collection of follow-up calls (6 months, 1, 2, and 3 years) will be completed in August 2023, February 2024, February 2025, and February 2026.

## Data Availability Statement

The original contributions presented in the study are included in the article, further inquiries can be directed to the corresponding author.

## Ethics Statement

Ethics approval to conduct this trial has been granted by the local Ethics Committee for the Region of North-western and Central Switzerland (EKNZ, Project-ID: 2019-00188). The latest amendment to the study protocol (version 6) has been approved by the EKNZ on 20th of June 2020. The trial will meet the criteria and principles of the Declaration of Helsinki and has been registered in the clinicaltrials.gov database (Trial registration number: NCT03184311, Registered 9th of June 2017).

## Author Contributions

BG, JK, RS, MB, SB-Z, ET, JL, HH, AS-T, and TD were broadly involved in the conception and design of the study and drafted the manuscript. Furthermore, BG, JK, and RS will be responsible for the logistic preparation and protocol-conform implementation of the study, the recruitment of patients, and the performance of training sessions. BG, JK, RS, AS-T, and TD critically reviewed the manuscript. The co-principal investigators of this study are AS-T and TD. TD and JL initiated the trial in collaboration with MB, SB-Z, ET, and AS-T. All authors have read and approved the final version of the manuscript and gave their consent for publishing this study protocol.

## Funding

This study is financially supported by the Swiss National Science Foundation (SNSF project No. 185217).

## Conflict of Interest

SB-Z is an employee of the funding institution (SNSF). However, her contribution to the study took place before her current employment at SNSF. The funder (SNSF) did not have any role in the study design, decision to publish, or preparation of the manuscript. The remaining authors declare that the research was conducted in the absence of any commercial or financial relationships that could be construed as a potential conflict of interest.

## Publisher's Note

All claims expressed in this article are solely those of the authors and do not necessarily represent those of their affiliated organizations, or those of the publisher, the editors and the reviewers. Any product that may be evaluated in this article, or claim that may be made by its manufacturer, is not guaranteed or endorsed by the publisher.
